# Multiplex gene editing to target the roots of hepatitis B virus infection

**DOI:** 10.1016/j.omtn.2026.103026

**Published:** 2026-08-03

**Authors:** Anuj Kumar, Fabien Zoulim

**Affiliations:** 1 Université Lyon 1, CNRS, ICBMS, UMR 5246, Villeurbanne, France; 2 The Lyon Hepatology Institute, IHU EVEREST, Lyon, France; 3 Université Lyon 1, Hospices Civils de Lyon, Inserm, PaThLiv, UMR 1350, Lyon, France

Chronic hepatitis B (CHB), caused by hepatitis B virus (HBV), remains a leading cause of hepatocarcinogenesis worldwide. The viral episomal covalently closed DNA (cccDNA) is the major driver of viral persistence. Although current nucleos(t)ide analogues (NAs) suppress viral replication, they fail to eliminate cccDNA, necessitating the need for a lifelong therapy for CHB patients. In the previous issue of *Molecular Therapy Nucleic Acids*, Slattery et al. demonstrate that multiplex CRISPR-Cas9 gene editing using *Staphylococcus aureus* Cas9 (SaCas9) and paired guide RNAs (gRNAs) reduces viral biomarkers and prevents viral rebound in preclinical HBV models.^1^ Importantly, this approach suppresses new random HBV DNA integration events into the host genome without increasing chromosomal translocations. While establishing long-term safety and delivery efficiency in clinical settings is important, these findings highlight a potential path to a functional cure.

With an estimated 240 million infected patients and over 1 million deaths annually, CHB remains a global health threat. In infected hepatocytes, cccDNA serves as the transcription template for all HBV RNAs and viral replication.^2^ HBV DNA can also integrate into the host genome generating HBV integrants, which are replication-incompetent but express HBsAg and truncated HBx. Integration also leads to the disruption of host genes and drive oncogenesis.^3^ While NAs efficiently suppress HBV replication, they do not directly eliminate cccDNA; hence, in virally suppressed patients who remain HBsAg positive, viral rebound is observed upon treatment discontinuation. Furthermore, functional cure, defined as sustained HBsAg loss and undetectable HBV DNA, allowing the safe cessation of antiviral treatment while maintaining long-term viral control, is rarely achieved in CHB patients under NA therapy.^4^

Gene editing technologies, such as CRISPR-Cas9, offer a direct approach to cleave and inactivate cccDNA. Several earlier studies have demonstrated the targeting of HBV DNA by CRISPR-Cas9, resulting in reductions of HBV markers.^5^ However, concerns about off-target effects, chromosomal translocations, and the risk that linearization of HBV DNA by Cas9 nuclease might elevate its integration into the host genome, a known driver of hepatocellular carcinoma, have hindered its clinical translation.^3^^,^^6^^,^^7^^,^^8^ While confirming the antiviral efficacy of CRISPR-Cas9-based multiplex gene editing, the work by Slattery et al. also attempts to assess its impact on HBV DNA integration as well as chromosomal instability.^1^

Using nonviral delivery of Cas9-encoding mRNA and HBV-targeting gRNAs, the authors targeted highly conserved sequences within the HBs/Pol and core-encoding regions. Using *in vitro* in primary human hepatocytes (PHHs) and *in vivo* in adeno-associated virus (AAV) and transgenic (Tg) HBV mouse models, the authors demonstrated marked inhibition of secreted HBV antigens (HBsAg and HBeAg) and intrahepatic HBV DNA levels. Hybridization-capture sequencing revealed the presence of indels, excisions, and inversions at the on-target sites in the viral genome, as well as a reduction in random HBV DNA integration. Importantly, the combination of CRISPR-Cas9 with standard-of-care NAs resulted in greater viral load suppression than monotherapy. From a safety perspective, no significant off-target editing at nominated human genomic sites was detected in PHHs, and no detectable increase in chromosomal translocations was observed in Tg-HBV mouse model, addressing two major concerns that have previously impeded the clinical translation of gene editing for CHB.

These findings hold substantial significance for the field. By targeting both cccDNA and integrated HBV DNA, the two primary templates for HBsAg production, this work provides preclinical evidence that CRISPR-Cas9-based approaches could pave the way toward a functional cure for CHB. Nevertheless, several limitations warrant consideration. Direct *in vivo* evidence of cccDNA editing is currently lacking, and the experimental models used do not fully recapitulate human CHB. For instance, AAV-HBV models lack true cccDNA formation, while Tg-HBV mice harbor artificial integration patterns that may not reflect the multiple and heterogeneous integration landscape observed in humans. Addressing these questions is challenging as pre-clinical models fully recapitulating the situation observed in chronically infected patients are still lacking. Moreover, long-term studies will be essential to exclude delayed off-target effects or chromosomal instability, and further investigation is needed to elucidate the mechanisms by which gene editing reduces HBV integration, as well as the fate of any residual unedited cccDNA. Additionally, the pathological implications of viral variant proteins that may arise post-gene editing remain to be determined. The current study also notes that only a few sequencing reads were assigned to translocation events, suggesting that further optimization of assays may be required to comprehensively assess this potential risk.

Moving forward, the clinical translation of these findings will depend on addressing several factors. Optimization of lipid nanoparticle delivery will be critical to ensure efficient and hepatocyte-targeted editing *in vivo*. Early phase gene and epigenome clinical trials, such as PBGENE-HBV (NCT06680232), TUNE-401 (NCT06671093), CRMA-1001(NCT07200193, EUCT 2025-523619-12-00), and EPI-003 (NCT06661148, NCT06745973) represent important milestones for the field, but long-term follow-up will be important to evaluate durability, safety, and efficacy in humans. Such studies will also help determine whether combination therapy will be needed to achieve high rates of functional cure.

Several challenges will need to be addressed to optimize antiviral efficacy and to prevent viral rebound if the reservoir of infected cells is not fully targeted and controlled. CHB is marked by profound dysfunction of HBV-specific B and T cell responses, which weakens immune-mediated clearance of infected hepatocytes.^9^ By lowering viral antigen burden through elimination or inactivation of viral genomic templates, gene editing could potentially create a more favorable environment for effective antiviral immune responses. Combining CRISPR-Cas9 with an immunomodulatory agent that boosts HBV-specific immunity, such as therapeutic vaccine or immune checkpoint inhibitor, may be beneficial. Additional combinations with entry inhibitors, capsid assembly modulators, antisense oligonucleotides (ASOs), or small interfering RNAs (siRNAs) may further enhance efficacy by targeting other components of the viral life cycle. The rationale for such combinations lies in their ability to target multiple aspects of HBV persistence simultaneously, hence increasing the likelihood of sustained HBsAg seroclearance and unquantifiable HBV DNA levels.^10^

Overall, the work by Slattery et al. provides an encouraging preclinical demonstration that multiplex CRISPR-Cas9-based gene editing can suppress HBV replication and integration without obvious chromosomal instability. While several challenges remain, these findings open new avenues for developing HBV curative therapies. As research advances toward clinical trials, integrating gene editing with complementary therapeutic strategies may ultimately unlock the potential for a functional cure, revolutionizing treatment for millions living with CHB.

## Acknowledgments

This work was performed in the frame of the IHU EVEREST (grant ANR-23-IAHU-0008), within the program “Investissements d’Avenir” operated by the ANR, and from the ANRS-EID HBV cure Task Force.

## Declaration of interests

F.Z. received research grant from Beam Therapeutics and had consulting activities for Precision Biosciences and nChromaBio.
